# Effects of controlled-release urea combined with fulvic acid on soil inorganic nitrogen, leaf senescence and yield of cotton

**DOI:** 10.1038/s41598-020-74218-2

**Published:** 2020-10-13

**Authors:** Jibiao Geng, Xiuyi Yang, Xianqi Huo, Jianqiu Chen, Shutong Lei, Hui Li, Ying Lang, Qianjin Liu

**Affiliations:** 1 Shandong Provincial Key Laboratory of Water and Soil Conservation and Environmental Protection, State Key Laboratory of Nutrition Resources Integrated Utilization, College of Agriculture and Forestry Science/Resources and Environment, Linyi University, Linyi, 276000 Shandong China; 2 Kingenta Ecological Engineering Group Co., Ltd, Linshu, 276700 Shandong China

**Keywords:** Physiology, Plant sciences, Environmental sciences

## Abstract

A split-plot field experiment was conducted in 2018–2019 to study the effects of nitrogen fertilizer types and fulvic acid (FA) rates on soil nitrogen and cotton growth. The nitrogen fertilizers included controlled-release urea (CRU) and urea, which were applied combined with three FA rates (90, 180 and 270 kg ha^-1^). The main plot was the nitrogen fertilizer type, and the subplot was the FA rate. The results showed that the lint yield of the FA180 treatment was 5.2–8.6% higher than the FA90 and FA270 treatments. Moreover, moderate FA application markedly improved the cotton leaf SPAD value (chlorophyll relative value), photosynthesis and chlorophyll fluorescence parameters compared with low and high FA rates. Replacing urea with CRU significantly increased the soil inorganic nitrogen and nitrogen use efficiency and also improved cotton fiber quality parameters. Meanwhile, the boll weight and seed yield of the CRU treatments were 1.5–8.4% and 3.3–19.1% higher, respectively, than the urea treatments. The interaction between nitrogen type and FA rate had a positive effect on cotton growth. Thus, the application of CRU combined with 180 kg ha^-1^ FA on cotton can not only improve the fiber quality and delay leaf senescence but also increase the yield and economic benefit.

## Introduction

Fertilizer is an important strategic material for ensuring food security and quality and is the material basis for maintaining agricultural capacity and realizing sustainable agricultural development^1^. China is a major producer and consumer of chemical fertilizer. The amount of nitrogen fertilizer used in China accounts for approximately 28% of the world’s total consumption, but the utilization rate of the nitrogen in the fertilizer is currently only 30–35%^2^. Moreover, the excessive application of nitrogen fertilizer leads to soil acidification, decreased organic matter content, deterioration of the soil physical and chemical properties, ammonia volatilization and nitrate leaching, which cause environmental problems^3,4^.

Cotton plays an important role in national production and is one of the most economically important crops and materials in China^5^. At present, China’s cotton planting areas are mainly divided into three dominant production areas: the middle and lower reaches of the Yangtze River, the Huanghuai Sea cotton area and the northwest inland cotton area^6^. However, in recent years, with the expansion of grain crops and vegetable planting areas, the size of the three major cotton planting areas has been significantly reduced. Therefore, the competition between cotton and other crops has become one of the important factors limiting the development of cotton production^7^. Appropriate fertilizer application is an effective way to increase the success of cotton farmers and resolve the competition between cotton and other crops.

Controlled-release urea (CRU) is a new type of fertilizer that releases nutrients according to a set mode through polymer coatings and other methods so that the nutrient release keeps pace with crop demand^8–10^. Compared with typical nitrogen fertilizer, CRU meets the needs of crops for nitrogen, effectively promotes the growth and development of crops, and simplifies cultivation technology. In addition, the agriculture ministry of the People's Republic of China has put forward an action plan with a goal of zero growth of fertilizer use by 2020^11^, which requires the demonstration and promotion of CRU and other new fertilization technologies.

The application of humic acid (HA) can substantially improve soil fertility and soil physical and chemical properties^12^. HA is a colloidal organic substance that increases the size of soil aggregates and makes the soil loose and able to absorb a large amount of water; the long-term use of HA significantly improves soil structure and properties^13^. Moreover, HA increases the effectiveness of chemical fertilizer, plays a synergetic role with chemical fertilizer, and reduces the adverse effects of chemical fertilizer on the physical and chemical properties of soil^14^. HA increases nitrogen fertilizer efficiency by reducing the loss of nitrogen volatilization, increasing the effect of urea significantly, and prolonging the effect of urea. Meanwhile, HA increases the absorption and utilization rate of nitrogen^15^. HA also increased the mineralization rate of organic nitrogen in the soil, which increased the content of soil available nitrogen.

Fulvic acid (FA) is the low molecular weight component of HA and is water-soluble. It not only increases the effects of seed dressing, root dipping and spraying but also stimulates root absorption^16^. FA can promote seed germination and increase the seedling growth rate, and has a particular promoting effect on the development of the crop root system, which stimulates the seedling to root quickly, grow more secondary roots, and increase the amount of roots, and increases the ability of the crop to absorb water and nutrients^17,18^. The stimulating effect of FA on the aboveground part of crops led to vigorous growth, taller plant height and a strong stem, especially in the early stage, and FA stimulates the physiological metabolism of plant cells when it is absorbed through plant roots, which is manifested in increases in respiration and photosynthesis^19^. The application of FA to the leaves of crops results in a reduction of stomatal opening and water transpiration, which might influence plant photosynthesis.

There are many studies on the effects of CRU application on cotton production worldwide^20–22^, but there is a lack of information about the application of CRU in combination with FA. Hence, the objective of this study was to investigate the effects of CRU combined with FA on (i) cotton yield and fiber quality; (ii) leaf senescence; (iii) soil inorganic nitrogen (NO_3_^–^N and NH_4_^+^-N) content; and (iv) nitrogen use efficiency.

## Results

### Leaf senescence in cotton

**Figure 1:**
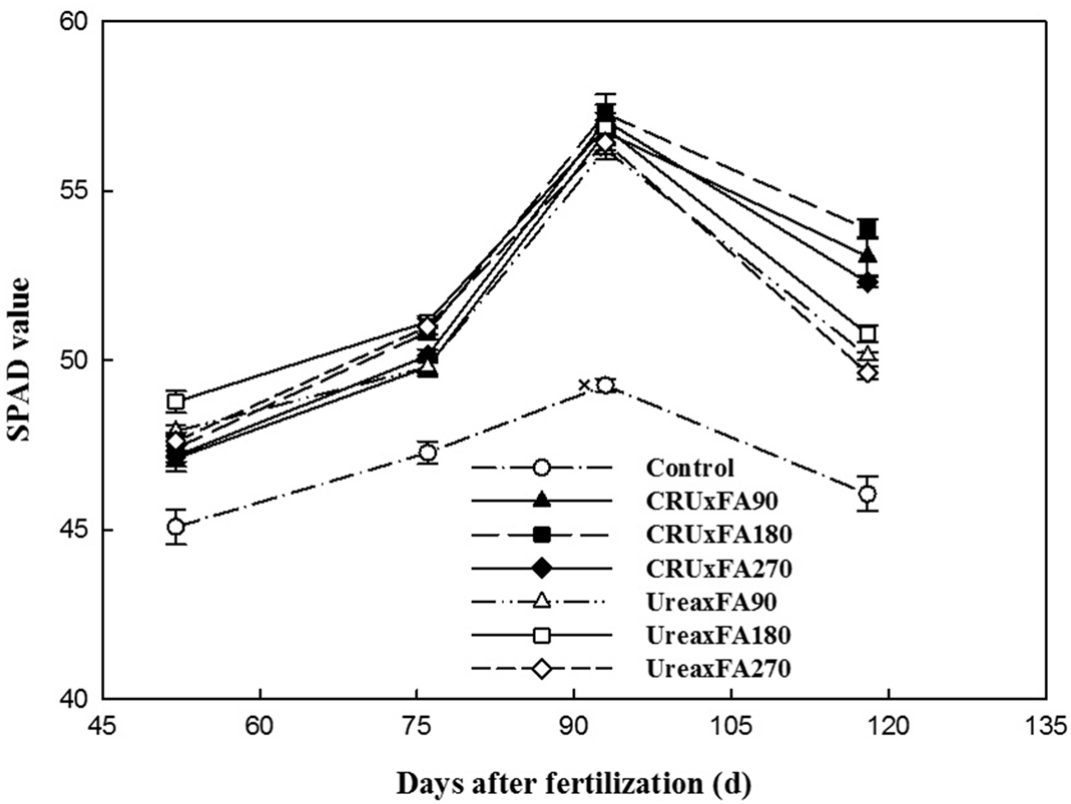
SPAD values. *Note*: SPAD value, chlorophyll relative value.

A series of physiological and molecular parameters were used to quantitatively measure leaf senescence symptoms. In this study, the SPAD value (chlorophyll relative value) of the Control treatment was the lowest among all the treatments in all growth stages of cotton (Fig. 1). At first, the SPAD value of the Urea treatments was higher than that of the CRU treatments, but after the first flowering stage, the SPAD value of the Urea treatments decreased 0.48–8.52%, rapidly. As the growing seasons went on, the SPAD value of the Urea treatments became lower than that of the CRU treatments. Otherwise, in both the Urea and the CRU treatments, the SPAD value of the 180 kg ha^-1^ fulvic acid (FA) treatment was higher than those of the 90 and 270 kg ha^-1^ FA treatments. The SPAD value of CRU × FA180 was the highest at the full boll-setting stage.

**Figure 2:**
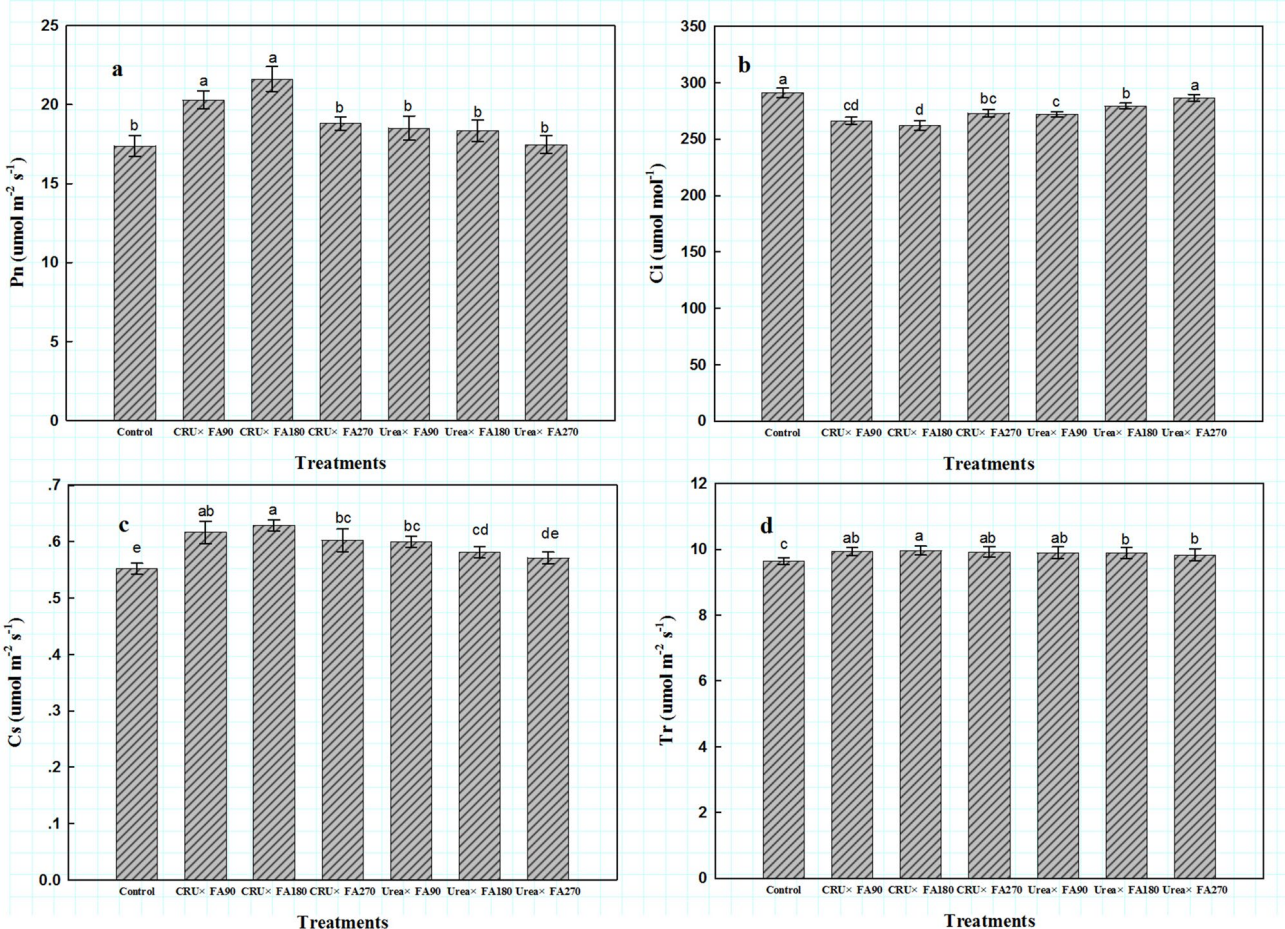
Photosynthesis indicators at full boll setting stages. *Note*: *P*_n_, photosynthetic parameters including net photosynthetic rate; *G*_s_, stomatal conductance; *C*_i_, intercellular carbon dioxide concentration; *T*_r_, transpiration rate.

**Figure 3:**
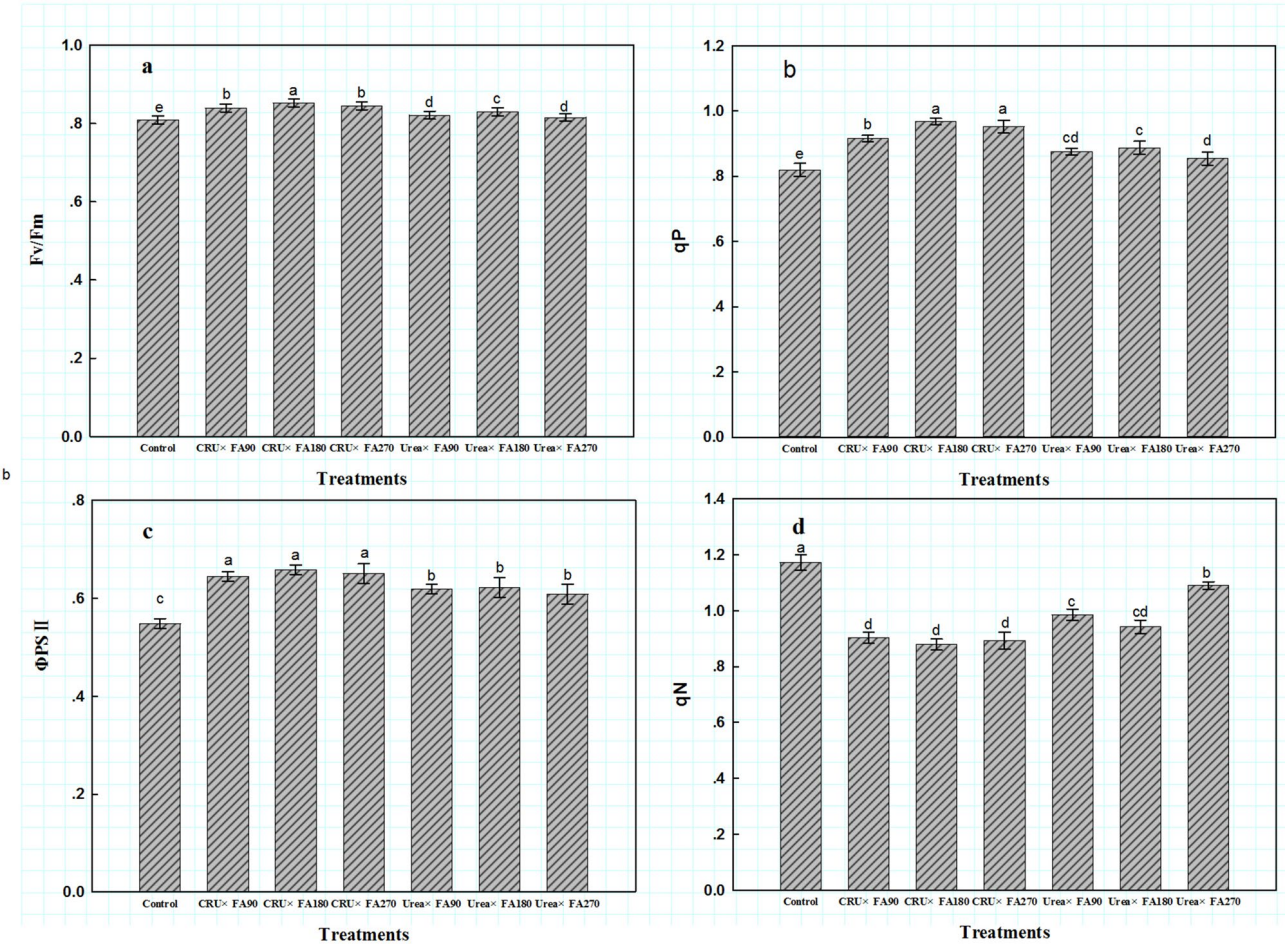
Chlorophyll fluorescence parameters at full boll setting stages. *Note*: *Φ*PSII, the effective quantum yield of PSII photochemistry; *F*_v_/*F*_m_, the primary light energy conversion efficiency; *q*_P_, photochemical quenching coefficient; *q*_N_, non-photochemical quenching coefficient.

Similarly, in the CRU treatments, the net photosynthetic rate (*P*_n_), stomatal conductance (*G*_s_) and transpiration rate (*T*_r_) were 11.70%, 6.89%, and 0.71% higher, respectively, but the intercellular carbon dioxide concentration (*C*_*i*_) of the CRU treatments was 4.54% lower than those in the Urea treatments (Fig. 2). In addition, the CRU treatment improved the effective quantum yield of PSII photochemistry (*Φ*PSII), the primary light energy conversion efficiency (*F*_v_/*F*_m_) and photochemical quenching coefficient (*q*_P_), but the non-photochemical quenching coefficient (*q*_N_) was lower than that in the Urea treatments (Fig. 3). Compared with the CRU × FA270 treatment, the CRU × FA180 and CRU × FA90 treatments markedly improved the Pn, Gs, and Fv/Fm and lowered the Ci, but no significant differences were found in Tr, Ci, PSII or qN among the CRU treatments. However, the amount of FA had no significant effect on photosynthesis or the chlorophyll fluorescence index of cotton leaves among the Urea treatments. Overall, the CRU × FA180 treatment greatly improved cotton leaf photosynthesis and delayed leaf senescence.

### Release characteristics of CRU

**Figure 4:**
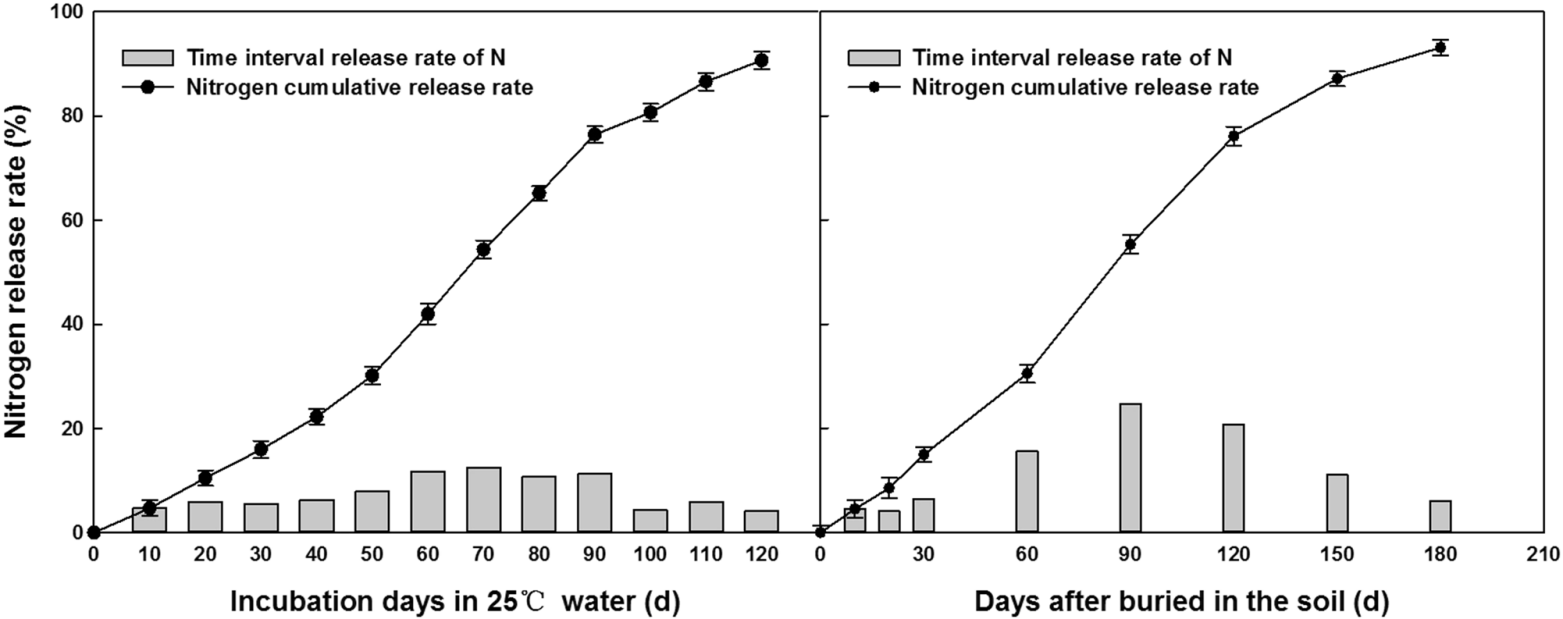
Release of nitrogen from CRU.

The nitrogen release curve of CRU showed a stable release rate (Fig. 4). Approximately 90.67% of the supplemental nitrogen was released in water at 25 °C by 120 days. The characteristics of nitrogen release in the field obviously slowed down, and only 76.12% of nitrogen was released within 120 days. The release of N from CRU in 25 °C water was mainly concentrated in the first 90 days. Under field conditions, nitrogen release can be maintained for 180 days. This meets the nitrogen demands of cotton, especially in the flowering and boll-setting stages.

### Soil inorganic nitrogen content

**Figure 5:**
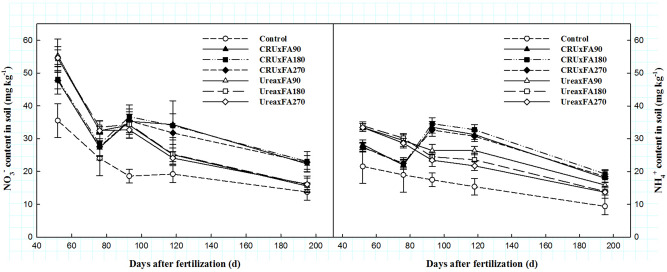
Change of NO_3_^–^N and NH_4_^+^-N content.

The soil NO_3_^–^N and NH_4_^+^-N contents under the Control treatment were the lowest of all the treatments throughout the whole cotton growing season (Fig. 5). With the increase in FA application, the content of NO_3_^–^N and NH_4_^+^-N in soil changed only slightly regardless of the type of nitrogen fertilizer applied. In the early growth stage, the contents of NO_3_^–^N and NH_4_^+^-N in the Urea treatments were higher than those in the CRU treatments, but after flowering, the contents of NO_3_^–^N and NH_4_^+^-N decreased rapidly, and the values in the Urea treatments were lower than those in the CRU treatments.

### Nitrogen uptake and nitrogen use efficiency

The nitrogen uptake, NUE and NAE of the CRU treatments were markedly higher than those of the Urea treatments (Table 1), but the dosage of the FA application had no significant effect. Specifically, the N uptake from the Control treatment was the lowest, and the N uptake from the CRU treatments was 2.03–2.58% higher than that from the Urea treatments. Similarly, the NUE and NAE of the CRU treatments were 7.45–9.59% and 9.30–15.48% higher, respectively, than those of the Urea treatments. In both the Urea and CRU treatments, there were no significant effects on N uptake or NAE regardless of the different FA dosages. However, the FA180 treatments markedly improved NUE compared with the NUE in the FA90 and FA 270 treatments. Generally, the CRU × FA180 treatment resulted in the highest N uptake and nitrogen use efficiency.

**Table 1:** Nitrogen uptake, nitrogen agronomy efficiency (NAE) and nitrogen use efficiency (NUE) of cotton plant after harvested in 2019.

Treatments	Nitrogen uptake (kg ha^-1^)	NAE (kg kg^-1^)	NUE (%)
**Nitrogen fertilizer type**			
Controlled-release urea (CRU)	262.11 a	9.51 a	37.54 a
Urea	256.78 b	8.53 b	34.60 b
**Fulvic acid (FA) rate (kg ha**^**-1**^**)**			
90	258.83 a	8.97 a	35.95 b
180	260.00 a	9.17 a	36.26 a
270	259.50 a	8.92 a	36.02 b
**Nitrogen fertilizer type × FA rate interaction**			
Control	194.2 c	-	-
CRU × FA90	261.3 a	9.4 a	37.5 a
CRU × FA180	262.7 a	9.7 a	37.7 a
CRU × FA270	262.5 a	9.5 a	37.5 a
Urea × FA90	256.4 b	8.5 b	34.4 b
Urea × FA180	257.8 b	8.6 b	34.9 b
Urea × FA270	256.1 b	8.4 b	34.5 b
**Source of variance**			
Nitrogen fertilizer type	< 0.0001	< 0.0001	< 0.0001
FA rate	0.3135	0.206	0.0072
Nitrogen fertilizer type × FA rate	0.898	0.87	0.2466

Means followed by different lowercase letters in the same column were significantly different based on analyses with ANOVAs followed by Duncan tests (*P* < 0.05).

### Cotton yields, fiber qualities and net profits

The type of nitrogen fertilizer, the amount of FA and their interaction all affected the boll number, boll weight and cotton yield (Table 2). The cotton seed and lint yields of the CRU treatments were significantly higher than those of the Urea treatments in the two consecutive years. Compared with those in the Urea treatments, the CRU treatments significantly increased lint yields by 4.73–18.12% in 2018 and 1.66–19.86% in 2019. In addition, the FA dosages markedly affected the cotton yields under the same fertilizer type. The lint yield of the CRU × FA180 treatment was the highest in both years, and there were no significant differences between the CRU × FA90 and CRU × FA270 treatments. Moreover, the average boll number and boll weight of the CRU treatments were 0.38–9.98% and 1.02–11.03% higher, respectively, compared with those in the Urea treatments. The Control had the lowest performance in each year. In addition, the lint percentage was not affected by the type of nitrogen fertilizer or the amount of FA applied. The lint percentage remained at 45.38–45.62% in the different treatments.

**Table 2:** Cotton yield and yield component under different treatments during 2018 and 2019 growing seasons.

Treatments	2018					2019				
	Bolls no. (m^2^)	Boll weight (g)	Seed cotton yield (kg ha^-1^)	Lint percentage (%)	Lint yield (kg ha^-1^)	Bolls no. (m^2^)	Boll weight (g)	Seed cotton yield (kg ha^-1^)	Lint percentage (%)	Lint yield (kg ha^-1^)
**Nitrogen fertilizer type**										
Controlled-release urea (CRU)	86.48 a	6.42 a	5549.94 a	45.50 a	2525.26 a	80.94 a	6.10 a	4940.04 a	45.63 a	2253.98 a
Urea	81.93 b	6.10 b	5002.70 b	45.51 a	2276.93 b	77.13 b	5.74 b	4431.00 b	45.56 a	2018.96 b
**Fulvic acid (FA) rate (kg ha**^**-1**^**)**										
90	83.80 b	6.20 b	5202.16 b	45.42 a	2362.97 b	77.93 b	5.78 c	4503.11 c	45.67 a	2056.51 c
180	85.72 a	6.37 a	5463.91 a	45.52 a	2486.90 a	80.88 a	6.05 a	4900.41 a	45.69 a	2234.07 a
270	83.10 b	6.21 b	5162.90 b	45.57 a	2352.97 b	78.30 b	5.94 b	4653.03 b	45.53 a	2118.83 b
**Nitrogen fertilizer type × FA rate interaction**										
Control	71.70 ed	5.60 d	4015.73 d	45.42 a	1824.13 d	72.07 d	5.61 d	4045.79 d	45.38 a	1836.31 d
CRU × FA90	85.72 ab	6.34 b	5435.08 ab	45.41 a	2468.44 ab	78.67 c	5.93 b	4664.98 b	45.69 a	2131.49 b
CRU × FA180	88.23 a	6.52 a	5752.64 a	45.49 a	2616.88 a	83.40 a	6.24 a	5203.74 a	45.57 a	2371.48 a
CRU × FA270	85.54 ab	6.39 b	5462.16 ab	45.59 a	2490.45 ab	80.77 b	6.13 a	4951.39 ab	45.62 a	2258.96 ab
Urea × FA90	81.97 bc	6.07 c	4969.25 c	45.42 a	2257.53 c	77.20 cd	5.62 d	4341.24 c	45.65 a	1981.54 c
Urea × FA180	83.23 b	6.22 bc	5175.22 b	45.54 a	2356.92 b	78.37 c	5.87 bc	4597.09 bc	45.61 a	2096.66 bc
Urea × FA270	80.70 c	6.03 c	4863.64 c	45.55 a	2215.49 c	75.83 cd	5.74 cd	4354.67 c	45.44 a	1978.69 c
**Source of variance**										
Nitrogen fertilizer type	< 0.0001	< 0.0001	< 0.0001	0.9547	< 0.0001	< 0.0001	< 0.0001	< 0.0001	0.5161	< 0.0001
FA rate	0.0004	0.0009	0.0002	0.6781	0.0009	0.0008	0.0017	< 0.0001	0.5011	0.0001
Nitrogen fertilizer type × FA rate	0.2877	0.3895	0.2851	0.9608	0.4161	0.0129	0.695	0.0251	0.6314	0.0323

Means followed by different lowercase letters in the same column were significantly different based on analyses with ANOVAs followed by Duncan tests (*P* < 0.05).

Compared with that in the Control, the application of N and FA fertilizer significantly improved the fiber quality (Table 3). The results showed that the fiber length, uniformity, strength and micronaire values of the PCU treatments were significantly higher than those of the Urea treatments. In addition, the fiber elongation and fiber quality were affected by the interaction of N × FA. Compared with those of the CRU × FA90 and CRU × FA270 treatments, the CRU × FA180 treatment significantly increased fiber length, uniformity, strength and micronaire value. Otherwise, there were no significant differences between CRU × FA90 and CRU × FA270, except in terms of fiber strength.

**Table 3:** Cotton fiber qualities under different treatments after two years’ fertilization (in 2019).

Treatments	Fiber length (mm)	Fiber uniformity (%)	Micronaire	Fiber elongation (%)	Fiber strength (cN tex^-1^)
**Nitrogen fertilizer type**					
Controlled-release urea (CRU)	27.3 a	83.7 a	5.5 a	6.8 a	27.8 a
Urea	27.1 b	83.3 b	5.4 b	6.8 a	26.9 b
**Fulvic acid (FA) rate (kg ha**^**-1**^**)**					
90	27.0 b	83.3 b	5.4 b	6.8 a	26.7 c
180	27.4 a	83.8 a	5.5 a	6.8 a	28.0 a
270	27.2 ab	83.4 b	5.4 b	6.8 a	27.4 b
**Nitrogen fertilizer type × FA rate interaction**					
Control	26.3 c	82.9 d	5.4 b	6.8 a	24.6 c
CRU × FA90	27.0 b	83.4 bc	5.5 ab	6.8 a	26.9 b
CRU × FA180	27.5 a	84.2 a	5.6 a	6.8 a	28.7 a
CRU × FA270	27.4 a	83.6 b	5.5 ab	6.8 a	27.8 ab
Urea × FA90	27.0 b	83.2 c	5.4 b	6.8 a	26.5 b
Urea × FA180	27.3 a	83.4 bc	5.5 ab	6.8 a	27.3 ab
Urea × FA270	27.0 b	83.2 c	5.4 b	6.8 a	27.0 b
**Source of variance**					
Nitrogen fertilizer type	0.0259	0.0002	0.0133	0.6075	0.0001
FA rate	0.0068	0.0005	0.0357	0.46	0.0002
Nitrogen fertilizer type × FA rate	0.3774	0.0114	0.683	0.7588	0.0485

Means followed by different lowercase letters in the same column were significantly different based on analyses with ANOVAs followed by Duncan tests (*P* < 0.05).

**Figure 6:**
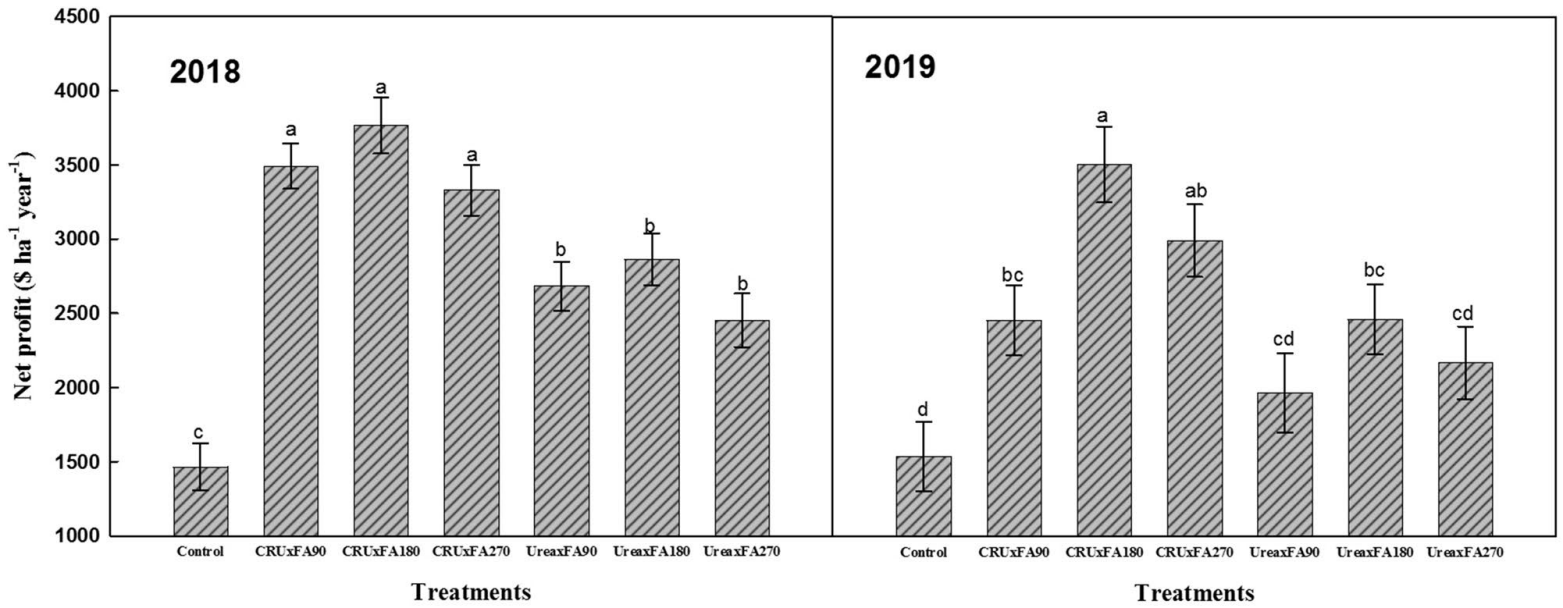
Mean net profits.

The net profit was calculated by the annual average income and costs (fertilizer, labor and other costs) of the different treatments (Fig. 6) in 2018 and 2019. The net profits ranged from 1466–3765 $ ha^-1^ year^-1^ and 1465–3150 $ ha^-1^ year^-1^, respectively. Compared with those of the Urea treatments, CRU treatments significantly improved the net profits. Moreover, the net profits from the middle FA rate treatments were better than those from the low and high FA rate treatments under the same nitrogen fertilizer type. The CRU × FA180 treatment resulted in the highest net profit, which was 7.9–53.6% higher in 2018 and 16.3–72.1% higher in 2019 than those of the other nitrogen treatments. In general, the CRU × FA180 treatment markedly improved cotton yields, fiber quality, and net profits.

## Discussion

### Cotton leaf senescence

The function of fulvic acid (FA) in agricultural production is mainly manifested in two aspects: one is to reduce the transpiration rate of crop leaves, which keeps more water in plants and soil, promotes root development, and makes crops absorb more water and nutrients^23^; the other is to increase the activity of various synthetases and chlorophyll content to maintain photosynthesis^24^. Over time, these functions of fulvic acid also serve to delay leaf senescence in crops. However, leaf senescence is the result of a variety of physiological changes. Generally, a single parameter cannot reflect leaf senescence but only reflects the specific physiological changes related to the measured parameter. In this study, the FA180 treatments showed higher SPAD value (chlorophyll relative value) and photosynthetic parameter values than the FA90 and FA270 treatments. In terms of nitrogen fertilizers, the CRU treatments might delay the senescence of cotton leaves, which was suggested by the higher photosynthesis index in the CRU treatments than in the Urea treatments. Therefore, the controlled-release nitrogen fertilizer effectively promoted the growth and development of cotton and improved the physiological characteristics of leaves; similar results have also been reported in other studies^20^. Therefore, based on its higher photosynthetic indexes, the CRU × FA180 treatment delayed leaf senescence.

### Soil inorganic nitrogen and nitrogen use efficiency

NO_3_^–^N is negatively charged and does not accumulate in the soil. It moves freely in the soil solution, so it can be absorbed by plants, but it can also be leached easily. NH_4_^+^-N can be adsorbed on minerals and organic matter, and it may compete with other cations that are important for plant nutrition^25^. However, FA can accelerate the mineralization of organic nitrogen in soil and increase the content of inorganic nitrogen in soil. In the present study, FA180 and FA90 increased the contents of soil NO_3_^–^N and NH_4_^+^-N compared with those under FA270 in both nitrogen fertilizer treatments, which means that only a suitable FA rate had positive effects on soil inorganic nitrogen. However, the rapid hydrolysis of urea causes serious nitrogen losses^26^. Thus, due to the continued nitrogen release by CRU, the contents of NO_3_^–^N and NH_4_^+^-N increased significantly compared to those in the Urea treatments from the early flowering to harvest stage, which was similar to the findings ^21^.

The main reasons for the high nitrogen use efficiency and nitrogen uptake might be that photosynthesis increased nitrogen transport to cotton bolls and decreased litterfall^27,28^. Regardless of the FA application rate, the nitrogen uptake in the CRU treatments was higher than that in the Urea treatments. Meanwhile, the NAE and NUE in the CRU treatments were higher than those in the Urea treatments^10^. The FA rate did not affect the nitrogen uptake or NAE, but it had significant effects on NUE; the FA180 treatment had a higher NUE than those of FA90 and FA270. Therefore, when the nitrogen supply is sufficient, FA application improves NUE. The CRU × FA180 treatment improved the inorganic nitrogen levels and NUE.

### Cotton yield and fiber quality

FA is a macromolecular organic compound of humic acid with a low molecular weight that contains many kinds of active functional groups and is easily absorbed by plants^29^. Some studies have also shown that the application of FA in cotton fields can reduce irrigation requirements, improve the growth of cotton plants, reduce the shedding of buds and bolls, and significantly improve cotton quality and increase yield^30^. In this study, the FA180 treatment markedly improved the boll number and boll weight, which led to higher seed and lint yields compared with those in the FA90 and FA270 treatments. In addition, the release characteristics of CRU provided enough nitrogen over the whole growth period to improve the seed yield and fertilizer utilization of cotton, which were better than those under urea. Specifically, the release rate of CRU was slow in the first month and accelerated from the early flowering stage to harvest stage. This pattern met the nitrogen absorption requirements for cotton growth. A similar result was also reported^20^. Moreover, there was a positive interaction effect between nitrogen type and FA rate, as shown by the cotton boll number and yields in 2019. Thus, the CRU × FA180 treatment increased the cotton yields and yield components.

Fiber quality is the decisive factor for cotton price. FA has been applied to dozens of crops, such as wheat, corn, sweet potato, rape and so on, and has shown obvious effects such as improved stress resistance, increased production and higher crop quality^31^. In the present study, the moderate FA rate treatments increased the fiber length, uniformity, strength and micronaire values in comparison with those in the low and high FA rate treatments, but no significant difference was found in fiber elongation. At the same time, the excessive application of nitrogen often leads to crop quality decline and higher disease susceptibility^32^. Compared with those in the Urea treatments, the CRU treatments increased fiber length, uniformity and strength, which might be due to the continuous supply of sufficient nitrogen from CRU during the critical growth period. However, neither nitrogen type nor FA application affected the fiber elongation of cotton, which might be more affected by genetic regulation than by fertilization. A significant nitrogen fertilizer type × FA rate interaction effect was found on fiber uniformity and fiber strength.

Overall, the type of nitrogen fertilizer, the FA rate and their interaction have a significant influence on cotton lint yield and fiber quality. The net profit and NUE of the CRU × FA180 treatment were 13.7–56.7% and 0.5–9.3% higher, respectively, than those of the other nitrogen treatments. Due to the continuous nitrogen supply from CRU, the contents of NO_3_^–^N and NH_4_^+^-N in the soil increased from the early flowering stage to the harvest stage, compared with urea. Cotton leaf photosynthesis improved with the appropriate FA rate application and CRU release traits. Therefore, it is suggested that CRU and 180 kg ha^-1^ FA should be used for cotton production to improve yield, fertilizer use efficiency and net profits.

## Materials and methods

### Experimental materials

The two-year experiment (2018–2019) was conducted at the Yinan County experimental base, Linyi City, Shandong Province, China (N 35°48′33″; E 118°26′45″). The climate is a temperate monsoon climate, and the precipitation is concentrated from July to September. The contents of sand, silt and clay in the soil are 547.32, 228.63 and 216.03 g kg^-1^, respectively, which are classified as sandy loam.

### Soil type and analysis

The soil type of the experimental site is classified as Typic Hapludalf according to the USDA classification^33^. The basic physical and chemical properties of the tested soil are listed in Table 4.

**Table 4:** Part properties of tested soil before cotton planting.

Year	pH value (2.5:1)	Organic matter (g kg^-1^)	Total N (g kg^-1^)	NO_3_^—^N (mg kg^-1^)	NH_4_^+^-N (mg kg^-1^)	Available P (mg kg^-1^)	Available K (mg kg^-1^)
2018	6.75	6.5	0.76	55.39	22.14	35.48	132.95
2019	6.70	7.2	0.88	58.21	25.92	37.04	133.57

### Fertilizer material

The tested fertilizers include controlled-release urea (CRU) and a typical urea fertilizer. The urea powder was poured into a disk granulator for granulation, and 3–5 mm particles were selected for drying. To make the CRU, after the surface of the particles was even and smooth, epoxy resin with a coating thickness of 4% was used to coat the particles. The CRU contained 442.4 g N kg^-1^, with a four-month release longevity in water. The typical fertilizers included fulvic acid (FA) (pure fulvic acid content 497.3 g kg^-1^), urea (N 457.6 g kg^-1^), potassium sulfate (K_2_O 494.4 g kg^-1^) and calcium superphosphate (P_2_O_5_ 145.2 g kg^-1^).

### Experimental design

In this study, a split-plot design with triple replication was used. The main plots were assigned the type of nitrogen fertilizer (controlled-release urea: CRU, typical urea: Urea); the subplots were assigned the fulvic acid (FA) rates of 90, 180 or 270 kg ha^-1^. No nitrogen was applied in control plots (Control). The subplot area was 30 m^2^ (5 m wide and 6 m long). All fertilizers were applied once before planting. The application rates were 180 kg ha^-1^ N, 90 kg ha^-1^ P_2_O_5_, and 180 kg ha^-1^ K_2_O. Other agronomic management measures were performed consistently in accordance with local farming practices.

Cotton was sown on April 26, 2018, and April 28, 2019. The tested cotton variety was “Lumianyan 28”, which was planted by double ridge mulching, with a large row spacing of 80 cm, a small row spacing of 60 cm, a plant spacing of 35 cm, and an average planting density of 50,000 plants ha^-1^. Before the experiment, nylon mesh bags with a width of 8 cm and a length of 10 cm were made. Ten grams of CRU particles were weighed and placed into the mesh bags, and the bags were sealed. In the CRU treatment area, a ditch (8 cm deep and 12 cm wide) was dug 10 cm away from one side of the sowing row, and 30 mesh fertilizer bags were laid on the bottom of the ditch. The fertilizer particles in the mesh bags were evenly spread to cover the soil in the ditch to determine the release characteristics of CRU in the field soil.

### Soil sampling and measurement

In 2019, soil plant samples were collected at the bud stage (52 days after sowing), the early flowering stage (76 days after sowing), the full boll-setting stage (93 days after sowing), the initial boll-opening stage (118 days after sowing) and the harvest stage (195 days after sowing). The soil samples were collected along a diagonal from five plow layers with a soil drill. After mixing, the samples were taken back to the laboratory for air drying and passed through a 10 mesh sieve. The screened samples were extracted with 0.01 M CaCl_2_, and the inorganic nitrogen (NO_3_^–^N and NH_4_^+^-N) contents were analyzed by an AA3-A001-02E automatic analyzer.

### Leaf sampling and analysis

The SPAD value (chlorophyll relative value), photosynthesis and chlorophyll fluorescence parameters were only measured at the full boll-setting stage. The SPAD value was measured by a hand-held chlorophyll meter (SPAD-502, Minolta, Japan). The net photosynthetic rate (*P*_n_), stomatal conductance (*G*_s_), intercellular carbon dioxide concentration (*C*_*i*_) and transpiration rate (*T*_r_) of the cotton leaves were measured using a LI-6400XT portable photosynthesis system (LiCOR, Lincoln, NE, USA). The primary light energy conversion efficiency (*F*_v_/*F*_m_), non-photochemical quenching coefficient (*q*_N_), photochemical quenching coefficient (*q*_P_) and effective quantum yield of PSII photochemistry (*Φ*PSII) were measured using an FMS2 portable fluorescence system (Hansatech Instruments, King’s Lynn, Norfolk, UK).

### Crop measurement

From the beginning of the boll-opening period, based on the boll setting situation, the total from 7 harvest times was assumed to be the yield of seed cotton in the plot, and this amount was converted to the yield per hectare. At the third harvest, 100 bolls in full bloom were collected, and the weight of a single boll was calculated; the number of bolls with a diameter greater than 2 cm was recorded as the number of bolls per plant. At the end of the harvest period, the aboveground parts of five cotton plants were collected, killed in an oven at 105 °C for 40 min, and dried at 70 °C to a constant weight. After grinding, the plant total nitrogen contents were determined by digestion of H_2_SO_4_-H_2_O and the micro-Kjeldahl method. The calculation methods for the nitrogen use efficiency (NUE) and nitrogen agronomic efficiency (NAE) were those described by Zhang^34,35^.

1. NUE (%) = (cumulative plant N uptake from N treatment- cumulative plant N uptake from no-N treatment)/the amount of N fertilizer applied × 100%;
2. NAE (kg N kg^-1^) = (the seed yield in the N treatment-the seed yield in the no-N treatment)/the amount of N fertilizer applied;
3. Net profit ($ ha^-1^ year^-1^) = the seed yield × cotton price-fertilizer costs-other costs-labor costs.

The mean prices of fertilizers and other nonlabor expenses in China (US dollars per ton) were set as CRU $348.4, urea $238.8, fulvic acid $214.6, potassium sulphate $540.1, calcium superphosphate $149.3, and cotton $1347.2. Other costs included machinery, plastic film, irrigation, pesticides, insecticides, seeds, and other materials and services at $721.0. The mean cost of labor in China was set as $12.80 for one employee day^-1^ ha^-1^.

### CRU release characteristics

The nitrogen release rate of CRU in water was determined by the national standard for slow release fertilizer of the People's Republic of China^36,37^. Ten grams of CRU was placed in a glass bottle containing 200 ml triple-distilled water and kept at a constant temperature (25 °C) in an incubator. The solution samples were collected at 10, 20, 30, 40, 50, 60, 70, 80, 90, 100, 110, and 120 days until the cumulative nitrogen release rate of the CRU (using the Kjeldahl method) was greater than 80%. Under field conditions, three mesh bags containing 10 g CRU were dug up at 10, 20, 30, 60, 90, 120, and 180 days and taken back to the laboratory. The soil was washed out with clear water, the net bag was cut open and the fertilizer was transferred to an aluminum box and dried to a constant weight. The “weight loss method” was used to determine the release amount, where the weight difference before and after the determination was regarded as the released amount of nitrogen.

### Statistical analyses

Microsoft Excel 2010 was used to preprocess the data, and SAS software (version 10, SAS Institute Cary, NC, USA, https://www.sas.com/en_us/software/university-edition.html) was used to conduct multiple comparisons with LSD, and ANOVA was conducted. All the data in this paper are the average values of three repetitions, and SigmaPlot software( version 12, MMIV, Systat Software Inc., San Jose, CA, USA, https://sigmaplot.en.softonic.com) was used to draw the figures.
